# Enhancing collaboration between national TB programmes and researchers

**DOI:** 10.5588/ijtldopen.25.0624

**Published:** 2026-02-11

**Authors:** L. Dall’Olio, P. de Colombani, J. Servello, G. Gargioni, R. Duarte, M. Raviglione

**Affiliations:** 1 Centre for Multidisciplinary Research in Health Science (MACH), Universita’ di Milano, Milan, Italy;; 2 EPIUnit ITR, Instituto de Saúde Pública da Universidade do Porto, Universidade do Porto, Porto, Portugal;; 3 Instituto de Ciências Biomédicas Abel Salazar, Universidade do Porto, Porto, Portugal;; 4 Centro de Saúde Pública Doutor Gonçalves Ferreira, Instituto Nacional de Saúde Doutor Ricardo Jorge, INSA Porto, Porto, Portugal;; 5 www.unite4TB.org.

**Keywords:** tuberculosis, collaboration, policy makers, clinical trials

Dear Editor,

The Università di Milano (UMIL), through its Centre for Multidisciplinary Research in Health Science (MACH), participates in the UNITE4TB consortium conducting phase 2 clinical trials (CTs) on new chemical entities and regimens for the treatment of TB. MACH focuses on engaging TB stakeholders by informing them about the progress of the CTs and obtaining their feedback for implementation and future prompt uptake of the innovations in countries. This approach is still relatively uncommon in TB research and development. Since 2022, MACH has been undertaking annual interviews with major TB stakeholders and participating in relevant global and regional events. In 2022, UMIL conducted a stakeholder analysis among 50 actors, including international policymakers, national TB programmes (NTPs), financial supporters, and organisations and agencies at national and international level. The analysis drew up 10 key recommendations for the continuation of the UNITE4TB project.^1^ Among these, emphasis was put on 1) enhancing engagement of NTPs in the conduct of CTs to ensure proper selection of sites and to facilitate local research capacity and 2) supporting NTPs in identifying bottlenecks for rapid uptake of new regimens. In the years 2023–2024, UMIL followed up on the above key recommendations with a selection of stakeholders and found that the engagement of NTPs by the CT principal investigators (PIs) was still a challenge and had potential for improvement. Of the 8 NTP managers consulted, only 3 reported good communication, whereas 5 felt insufficiently informed about the ongoing CTs.

Given the crucial role of NTPs in the implementation of CTs and uptake of innovations, UMIL designed a survey to identify key elements for effective PIs–NTPs communication and collaboration. The survey targeted PIs and NTP managers from the countries participating in the PARADIGM4TB trial, which were already recruiting (in South Africa, Tanzania, and Uganda), or were about to start (in Moldova and Viet Nam). Participants were invited by email to fill in an online questionnaire developed in Microsoft Forms and containing a total of 16 questions, both closed and open-ended, on practices and behaviours. The questionnaire was initially tested and then distributed on 20 February 2025, allowing 2 weeks to respond. Four reminder emails were also sent before closing the response period on 2 April 2025. The survey was approved by the Ethics Committee of the Università di Milano.

Only 2 out of 5 NTP managers (40%) and 7 out of 13 PIs (54%) contacted completed the questionnaire. Within a proposed ideal list of conditions for effective communication/collaboration, the NTP managers identified ‘Having clear guidelines and communication means’ and ‘Having clinical trial aligned with the national research plan’ as the most important; the PIs indicated ‘Having clear guidelines and communication means’ and ‘Having good personal relation with counterpart’. The least important condition for both NTP managers and PIs was ‘Working in the same building’. Investigating and comparing past/current and desired future collaboration areas, the questionnaire revealed a clear intention to broaden the scope of collaboration across nearly all proposed areas. While this was expected from the NTP managers, it was also expressed by the PIs and in relation to both CT implementation (engagement of communities and civil society organisations, selection of people to screen, information and education, drug intake supervision and support, management of people lost to follow-up) and, surprisingly, to CT management (designing/planning CTs, advocacy with political authorities, pharmacovigilance) (see Figure).

**Figure.**
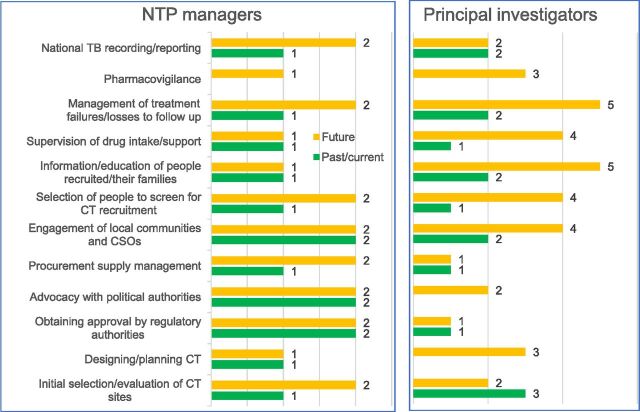
Areas of collaboration between principal investigators and national TB programmes (NTPs) in the past/current, and desired in the future by type of respondent. CSO = civil society organisation; CT = clinical trial.

This study, although limited in scale, provides an initial insight into the operational challenge of implementing CTs while considering future uptake of TB innovations in countries.

Since the start of UNITE4TB, UMIL’s stakeholder analysis and engagement have fostered conditions for the timely adoption of TB innovations. The multi-year process of interviews, dialogue, and policy reflection has advanced the recommendations, particularly by strengthening collaboration between researchers and NTPs, through ongoing follow-up, constructive pressure, and fine-tuning. However, survey participation was low, especially among the NTP managers, likely due to the limited number of countries and individuals involved, the concerns about being identified, and the reluctance to share negative views. This is disappointing given how often NTP managers report inadequate communication with researchers and their institutions. The insights of the survey confirm that collaboration between researchers and NTPs is thriving where interaction is early, structured, and multifaceted, but remains fragile otherwise. Strengthening these partnerships depends less on the frequency of contact than on broadening their scope, clarifying roles and equipping teams with the means to work together. The following actions are proposed as immediate steps to strengthen collaboration between NTPs and research institutions at country level:

⁃ CT coordinators should systematically include NTPs in their agendas during country visits.
⁃ CT researchers (coordinators and field investigators) should work with NTPs to develop or revise CT implementation plans that specify collaboration areas and responsibilities, communication means and tools, monitoring mechanisms, and required training.
⁃ CT researchers should systematically engage key TB stakeholders and conduct ad hoc surveys to assess and monitor PIs–NTPs communication and collaboration.

Although limited by size and potential response bias, our survey echoes persistent concerns identified in earlier analysis.^2^ The consistency of these signals reinforces their validity and suggests practical measures to enhance PIs–NTPs collaboration. The systematic adoption of these measures reinforces the proposed standards for implementing CTs^3^ and is instrumental for their success, while integrating and building national research capacities and preparing for the rapid adoption of innovations in national policies and strategies. The protocols of future CTs should describe means and tools for PIs–NTPs collaboration and be submitted for authorisation and ethical clearance by the national authorities.
